# Nutrition Knowledge of Collegiate Athletes in the United States and the Impact of Sports Dietitians on Related Outcomes: A Narrative Review

**DOI:** 10.3390/nu13061772

**Published:** 2021-05-22

**Authors:** Aaron J. Riviere, Rae Leach, Haleigh Mann, Samuel Robinson, Donna O. Burnett, Jeganathan R. Babu, Andrew Dandridge Frugé

**Affiliations:** 1 Department of Education and Human Development, Texas A&M University, College Station, TX 77843, USA; 2 Department of Nutrition, Dietetics, and Hospitality Management, Auburn University, Auburn, AL 36849, USA; rnl0006@auburn.edu (R.L.); hmm0041@auburn.edu (H.M.); dob0002@auburn.edu (D.O.B.); jeganrb@auburn.edu (J.R.B.); fruge@auburn.edu (A.D.F.); 3 Advent Health, Tampa, FL 33613, USA; srobinsonx23@gmail.com

**Keywords:** student athlete, sports nutrition, sports dietitian, college athletics

## Abstract

In the last decade, the number of full-time registered dietitians (RDs) serving intercollegiate athletes in the United States has more than quadrupled. However, many student athletes may be at increased risk of nutrition-related problems that impact physical and academic performance, which include inadequate macronutrients, inadequate micronutrients, and excessive macronutrients. This narrative review reports the current literature to date on nutrition-related knowledge in collegiate athletes and the impact of sports RDs on student athletes’ nutrition knowledge and behaviors. To date, only observational and quasi-experimental studies have been published with regard to changes in nutrition knowledge and behaviors in NCAA athletes. While these studies report benefits of the RD as a member of the interdisciplinary student athlete support team, more well-designed randomized control trials are warranted to determine benefits related to health outcomes and sport-specific performance outcomes.

## 1. Introduction

The first full-time registered dietitian (RD) was hired by an American university athletic department in 1994 [1]. The Collegiate and Professional Sports Dietitians Association (CPSDA) was chartered in 2010 to provide continuing education for and advancement of sports RDs. Since the National Collegiate Athletic Association’s (NCAA) deregulation of feeding in 2014, the number of sports RDs in the collegiate setting has grown exponentially to meet the new demand [2]. Over the past decade, studies have been conducted to assess nutrition needs and problems of collegiate athletes. These problems can be summarized in three categories: inadequate macronutrients, inadequate micronutrients, and excessive macronutrients.

### 1.1. Inadequate Macronutrition

A wide variety of nutrition-related issues commonly affect student athletes across the spectrum of body composition and type of sport. Low energy availability (EA), defined as an inadequate amount of dietary energy remaining for physiological function after exercise, is commonly seen in all categories of student athletes [3]. Low EA may arise from either an intentional or unintentional caloric deficit and is often accompanied by an athlete’s lack of knowledge about their nutrition needs. Athletes with relative energy deficiency in sport (RED-S) are in prolonged states of low EA and are at increased risk for infection, illness, fatigue, and nutrient deficiencies, as well as musculoskeletal, endocrine, gastrointestinal, renal, psychological, cardiovascular, and performance detriments. Athletes present with altered hormone levels, unfavorable body composition, catabolic markers, and menstrual dysfunction in females when large energy deficits are maintained long-term [3,4]. Poor carbohydrate, iron, and calcium intakes are commonly observed, especially among female athletes [5]. Likewise, as many as 80% of competitively trained male endurance athletes may be at risk for low EA [6], which is accompanied by impaired athletic performance, fatigue, basal metabolic rate, mood, and higher visceral fat deposition as well as increased risk for dehydration, injury, and illness [7]. The total energy needs for female volleyball players can be as high as 2815 kcals (±306) per day, or 39 to 44 kcal/kg of body weight, to support their higher percentage of lean mass [8]. Many female athletes consciously or unconsciously restrict their intake of carbohydrate below the recommended range of 6–10 g/kg of body weight, or what is needed for the maintenance of glycogen stores [9]. It is important for proper nutrition intake to be completed so that the current energy and nutrient consumption can be assessed, and changes can be made. These methods include 24-h recalls, food frequency questionnaires, diet history, and food records. Twenty-four-hour recalls can either be completed as a single day or for multiple days. The accuracy of this method is improved by proper training and using a multi-pass system that makes sure to catch any forgotten foods and drinks. Food frequency questionnaires look at trends in usual intake instead of single days individually. These are better for addressing whole-team diet trends and may under-estimate individual differences in dietary patterns. Diet histories are conducted by trained professionals who ask about typical diet patterns and behaviors that are associated with the diet pattern. These may include clinical symptoms, sleep, supplement, lifestyle, and exercise factors that may influence or be influenced by the individual’s diet. A final way for nutrient assessment is food records. These are written journals of everything the individual eats and drinks throughout the day and can include weights of foods and more details than other methods. Food records often require training of the individual on measuring food quantities and individuals may alter diet patterns since they are more aware of diet choices and the nutrients associated with each food [10].

Eating disorders are common in athletic populations, with recent estimates indicating as many as half of all athletes in aesthetic-focused sports, such as track and field, may present with features of disordered eating [11]. The gender gap in eating disorder risk may be narrower in athletic populations than in normal populations, with as many as 19% of male college athletes presenting with symptomology of a clinically relevant eating disorder [12,13]. A study of 111 male student athletes found previous food insecurity was associated with disordered eating behaviors such as preoccupation with food and hiding food in athletic locker rooms [14]. Disordered eating that results in insufficient dietary intakes may be associated with additional negative physical consequences similar to RED-S and may include symptoms such as urinary incontinence in female athletes [15].

### 1.2. Inadequate Micronutrition

Outside of micronutrient deficiencies associated with inadequate energy intake, several micronutrients have direct correlations with athletic health and performance. Research is limited on ideal micronutrient intakes, but many micronutrients have the potential to impact sports performance if they are deficient. Thiamin, riboflavin, niacin, and pyridoxine all have important roles in energy status and metabolism within the cell. Since athletes have higher metabolic need and increased energy usage, they may see performance decrements even at subclinical levels. B12 and folic acid aid in hemoglobin formation while vitamins A, C, and E are all crucial for reducing oxidative damage. These vitamins all could decrease endurance performance if subclinical deficiencies exist [16]. Adequate iron status and fitness have been observed to yield combined positive effects on performance in classes [17]. The prevalence of iron deficiency is greater in athletes and active people, with occurrence rates in 20–50% in female athletes and 4–50% in male athletes [18,19]. Vitamin D deficiencies are common in student athletes, especially in indoor sports and during winter months when sun exposure is limited. Halliday et al. found that supplementation could play a role in disease prevention for college athletes. Further research may support nutrition’s role in cognition and injury prevention [20].

### 1.3. Excessive Macronutrition

Sport-specific events and positions, such as American football linemen and track and field throwers, attain mechanical advantages by maximizing their body size. The deliberate accumulation of large amounts of body mass, with body weights often exceeding 300 pounds, may place undue strain on the heart [7,21,22]. Several longitudinal studies have observed concentric left ventricular hypertrophy among football players, with mortality due to hypertensive heart disease and coronary artery disease increased by more than half (52%) among former football linemen [17,18]. Former football players with a BMI greater than 30 during their years in sport had close to double the risk of CVD mortality of other players (hazard ratio: 2.02 [95% CI, 1.06–3.85]) [23]. Other research studies have included metabolic syndrome (MetS) in order to investigate both cardiovascular risk and other factors to see the greater picture of the health of college football players. Linemen were more likely to meet MetS criteria in both high school and college settings than any other position or skill player. Higher body fat levels were also predictive of arterial blood pressure, HDL, and waist circumference [24].

Given the previously outlined nutrition-related problems in collegiate athletes, we sought to answer the following questions through this narrative review of the published literature: “Are current nutrition knowledge and practices among NCAA student athletes sufficient to meet established needs?” and “Do sports RDs positively impact nutrition knowledge and behavior in NCAA student athletes?”.

## 2. Materials and Methods

A narrative review process was selected due to insufficient studies examining the impact of sports dietitians on the health and performance of student athletes. The narrative review allowed for an evaluation of the limited research while showing the extensive literature base on the nutrition deficits and needs of this population. The literature search was conducted through the PUBMED and Web of Science databases by combining the keywords (dietitian AND athlete) OR (athlete AND nutrition AND knowledge) through 31 March 2021. The search results were limited to human studies that were written in English and conducted in the past ten years. The participants in the studies were required to be collegiate athletes in the United States.

## 3. Results

### 3.1. Nutrition Knowledge Needs of Student Athletes

One hundred twenty-one papers and 253 papers were retrieved from the (dietitian and athlete) and (athlete and nutrition and knowledge) searches, respectively. One hundred sixty-one and 72 were excluded due to geographic location, and the remaining exclusions were due to the wrong study population and/or not an observational or interventional study. The remaining fourteen studies are presented as assessing nutrition knowledge (Table 1, *n* = 8) or reporting the efficacy of dietitians (Table 2, *n* = 6).

In a 2016 study by Andrews et al., 123 student athletes from a Division I (DI) university with no sports dietitian on staff completed a questionnaire to determine their sports nutrition knowledge. While a predetermined mean score of 75% represented adequate knowledge, the mean score for this group was 56.9%. Only 12 student athletes out of the group scored 75% or higher, and there were no differences by team, class level, gender, or completion of prior nutrition coursework [25]. A group of 77 male and female college-aged indoor or sitting Paralympic volleyball players were given the sports nutrition knowledge questionnaire (SNKQ) developed by Zinn, Schofield, and Wall (2005). The mean raw score on the SNKQ of this group was 40.22 ± 8.39 out of a possible 88.00, a percentile score of 46 ± 9%. No athlete made the established passing score of greater than 70% on the quiz [26].

Madrigal et al. surveyed 196 student athletes about their nutrition knowledge and regrets over past eating and drinking habits. The strongest regrets were related to diet quality and inadequate fueling. Average scores were 48% for males and 49% for females and less than 25% for questions related to supplements, all of which were below the minimum values representing adequate knowledge. There was no association between nutrition knowledge and nutrition-related regrets, suggesting that even with the knowledge that they do have, student athletes need more help with behavior change [27]. Shriver et al. assessed the dietary intakes and eating habits of NCAA DI female college athletes and found that there were several sports nutrition standards that most were not meeting. The total calorie and carbohydrate intakes were below recommended minimums (*p* < 0.001), with only 9% meeting their daily energy needs and only 25% consuming the minimum amount of carbohydrate required for training. In addition, the vast majority (73%) did not eat a regular breakfast, and only 16% monitored their hydration status [28].

Brown et al.’s research suggests that football teams have a high degree of interest in working with a sports dietitian. One study assessed the nutritional needs of a DI football team who did not have an RD or training table, as well as their attitudes towards expanding the provision of nutrition services. Sixty-seven football players (90% of the team) completed a questionnaire, and their fueling and hydration strategies were noted to be insufficient for promoting optimal performance. A majority of the team (80–92%) did not comply with well-known nutrition strategies for football teams (92% did not consume a pre-game snack, 88% did not consume a pre-practice snack, 80% only drank water during exercise), and 75% reported a lack of energy during workouts. Interest in food and nutrition assistance was widespread, as 93.4% believed that they would benefit from a training table, 91% would be willing to meet with a dietitian, and 97% believed that proper nutrition would enhance their football performance [29].

In a similar study with DIII football athletes, a mean score of 55.2% on a nutrition knowledge questionnaire showed a poor understanding of nutrition. Less than half of the athletes consumed a daily fruit and vegetable while lineman had the greatest health-adverse dietary choices. Athletes that had been exposed to nutrition education through coursework had significantly higher results than the others [30].

A study of DIII student athletes at multiple universities examined energy drink consumption and nutrition knowledge, comparing these factors with GPA and team to determine the association between student athlete knowledge and practices. The results included a negative correlation between energy drink use and nutrition knowledge, and also between energy drink use and GPA [31]. Barrack et al. found that nearly half of a group of 557 NCAA DI athletes reported habitual supplement use [32]. It is estimated that 12–58% of all dietary supplements marketed for sport and exercise performance may contain ingredients prohibited by the World Anti-Doping Code (WADC) which may lead to halted sport participation [38]. Osterman et al. surveyed 307 male and female NCAA DI athletes about supplement knowledge needs, and found that athletes with the least perceived knowledge were more likely than those with greater perceived knowledge to list a question related to supplement quality or composition (*p* = 0.030) [39].

### 3.2. Efficacy of Sports Dietitians

Few studies to date have directly assessed the efficacy of the sports RD in the collegiate setting, particularly randomized control trials with an analysis of causal relationships (Table 2). Rossi et al. performed a matched control study with D1 baseball players. Participants were given a sports nutrition education intervention (SNEI) and compared to position-matched teammates. They found that intakes of kilocalories, protein, and carbohydrates increased significantly (*p* < 0.001) post-nutrition intervention in the SNEI group though body weight increases were similar between groups. In the intervention group, body fat percentage decreased significantly (*p* = 0.010), while the control group had a nonsignificant increase. The only performance outcome improved by the intervention was the 5-10-5 shuttle test (*p* = 0.030). This study showed an overall body composition improvement and a single performance outcome improvement when implementing an off-season nutrition intervention [33].

Both coaches and athletes are often unaware of the inadequate calorie and macronutrient intakes that are common among female athletes [11]. Valliant et al. reported findings in which volleyball players received individualized nutrition education from a sports RD over a four-month period, with a focus on increasing the knowledge of types and amounts of foods specific to personal dietary needs and activity level. The athletes completed a sports nutrition knowledge survey at the beginning and end of the intervention. Data from the non-intervention period indicated that athletes did not meet the recommended energy requirement of 37–41 kcal/kg. At the start of the intervention season, the participants consumed 56% of their estimated energy needs, with a range of 25–88%. At the end of the intervention season, the average intake rose to 70% with a range of 44–95%, representing a significant improvement (*p* = 0.002). The team’s average carbohydrate intake also fell short of the recommended 6–10 g/kg, with an average of 48% of estimated needs at the beginning of the intervention season, and a range of 29–76%. After the intervention, the average carbohydrate intake rose to 66% with a range of 33–101%, a significant improvement (*p* = 0.010). The average protein intake at the beginning of the intervention season was 59% of the estimated needs, with a range of 16–88%; at the end, it improved significantly (*p* = 0.010) to 72% of needed protein with a range of 37–102%. Nutrition knowledge improved significantly across the intervention (*p* = 0.001), with every participant answering more questions accurately on the post-test than they did on the pre-test. The mean scores were 24.7 (±5.9) for the pre-test and 31.5 (±6.1) for the post-test, out of a possible 55 points [8].

In a case study of a DI track and field female athlete, nutrition counseling was shown to positively affect aspects of the female athlete triad. The athlete presented with an energy deficit and menstrual dysfunction while showing continued weight loss prior to the nutrition program. After the one-month nutrition counseling program, weight loss stopped, body fat percentage increased, and performance was not negatively impacted over a 16-month follow up period [34].

At two universities that employed sports RDs, 383 student athletes were surveyed about their sports nutrition knowledge and habits, as well as about their primary source of nutrition information. When student athletes indicated the sports RD as their primary source, they demonstrated a greater understanding of nutrition periodization (47.12% vs. 32.85%), were more likely to consume school-provided boxed meals while on team trips (21.29% vs. 6.77%) and were less likely to consume fast food while on the road (9.90% vs. 19.55%). It was also noted that female athletes tended to eat more nutritiously than male athletes—they were more likely to prepare their own meals, eat breakfast 7 days a week, and consume school-provided boxed meals while on team trips. Male athletes, on the other hand, ate more fast food and restaurant meals, and had more frequent and higher weekly alcohol intake during their competitive seasons [1].

One study investigated nutrition behaviors in NCAA DI baseball players from three universities. Two universities employed a full-time sports RD (schools 1 and 2), while a third (school 3) had no sports RD on staff. All universities had a Certified Strength and Conditioning Specialist (CSCS) on staff, who tended to serve as the primary source of nutrition information at school 3. The majority of athletes sought individual nutrition counseling at schools 1 (61%) and 2 (53%), while those who did not seek personalized assistance utilized team services. Surveys indicated significant differences in dietary habits between student athletes who worked with a sports RD and those who did not. More student athletes from schools 1 and 2 reported never consuming fast food (31% vs. 7%, *p* = 0.020) or soda on a weekend day (50% vs. 26%, *p* = 0.080). The RD groups were also more likely to take a multi-vitamin daily (56% vs. 26%, *p* = 0.020) and consume fast food less frequently on team trips (45% vs. 70%, *p* = 0.010). The group who primarily received nutrition information from a strength and conditioning coach ate more frequently at hamburger restaurants (21% vs. 6%, *p* = 0.020), while the schools with a sports RD were more likely to have meals pre-planned when traveling on team trips (48% vs. 13%, *p* = 0.010). School 3 reported sport coaches who were less aware of healthy food options for the team when traveling (42% vs. 27%, *p* = 0.050). Regarding nutrient timing, there were also significant differences in responses between groups. Student athletes at schools 1 and 2 found it easier to eat within 1–2 h of activity (92% vs. 67%, *p* = 0.030), were more likely to have a pre-workout breakfast (67% vs. 38%, *p* = 0.020), and were more likely to prepare three or more meals on their own each week (86% vs. 74%, *p* = 0.070). Overall, the availability of a sports RD was correlated with the presence of positive nutritional habits in NCAA DI baseball athletes [35].

Sports RDs do not only have the role of educating student athletes, but they also have a role in training other staff members [36]. Torres-McGehee et al. assessed nutrition knowledge in multiple staff groups as well as with student athletes. They found most collegiate coaches and athletes do not have adequate nutrition knowledge, while some athletic trainers and strength and conditioning specialists may have adequate knowledge. Since these staff groups have daily interactions with the athletes, unlike many sports RDs, the authors discussed how proper nutrition training for all of these groups by a nutrition professional could have a major impact [36]. Evidence suggests that nutrition education programs should be taught by qualified nutrition professionals in order to be effective due to extensive training and knowledge. Zuniga et al. observed that most collegiate athletes use non-nutrition professionals as their sources for nutrition information but would be interested in using mobile applications for nutrition information [37]. These apps could be designed and maintained by nutrition professionals, which would allow for daily interactions by these trained professionals and further increase the use of their services by student athletes.

## 4. Discussion

Current observational and quasi-experimental studies suggest that sports nutrition knowledge among NCAA collegiate athletes is poor and does not meet current recommendations [25,26,27,28,30]. These changes have the potential to decrease athletes’ risks for issues including chronic energy deficits and injury, disordered eating behaviors, and long-term metabolic and cardiovascular risks [3,4,5,6,7,8,9,10,11,12,13,14,15,16,17,18,19,20,21,22,23]. Spronk et al. found that there is a positive correlation between nutrition knowledge and eating habits [40]. This correlation has also been shown in collegiate football players, though more populations need to be studied to assess if this is seen across all sports [30]. With this clear need for nutrition intervention, the next step is to find a solution to improve the performance and health of the athletes. Athletes often look towards their coaching and athletic staff to fill this knowledge gap. Though these staff members are experts in their respective fields, they often lack the needed nutrition knowledge to address the deficits and needs of the athletes, as shown in the systematic review by Trakman et al. [41]. A solution to this lack of nutrition expert is the addition of a sports RD. Sports RDs in the collegiate setting have increased the nutrition knowledge of student athletes, as well as improved their dietary intake [8,33,34,35]. As stated in the introduction, basic tasks for understanding current nutrition assessment requires trained professionals to be able to interview the athlete and properly record all foods consumed that may be missed by untrained professionals. Many institutions across collegiate and professional settings have already implemented some level of nutrition support, but there are still numerous universities that lack a nutrition professional to help their athletes. According to the Collegiate and Professional Sports Dietitians Association, 61 of the 65 Power Five schools (the five largest conferences in college athletics (ACC, Big Ten, Big 12, Pac-12, SEC)) have at least one sports dietitian while only 31 schools outside of the Power 5 schools have a sports dietitian on staff [42]. This means that thousands of student athletes are left without proper nutrition support. This review supports the benefit of a sports dietitian for all student athletes in order to meet their nutrition needs. Universities can start with contracting a dietitian for part time work to assess what needs are present and develop a plan for the university.

Though the general benefit of a sports RD has been shown in numerous observational studies as discussed in this review, well-designed studies are warranted to better determine the effects of the RD on nutrition knowledge, behaviors, academic performance, and sport performance. Assessment of the effect of the RD on injury risk and time to recovery may be most beneficial to universities seeking to justify the addition of one or more full-time RDs to their student athlete support staff. Many universities have introduced dining facilities, training tables, and many other nutrition-related improvements at once, so it may be difficult to identify which of these factors provides the most benefits to student athletes. More research is needed on the effect of the sports RD separate from other behaviors to determine best practices to increase their effectiveness. Though these other nutrition support options can help the athletes, a sports RD is needed on staff in order to ensure the training table is meeting each athlete’s needs and nutrition information spread through these other options, such as social media, is adequate and appropriate [43]. There has been a lack of structure for how a sports dietitian aids their athletes due to the recent creation of the position, but there have been efforts by the professionals in the field to further standardize their practice [44]. This will further increase the benefits of the sports RD on staff as well as help universities validate the position by looking at how the sports RD is benefiting similar schools and programs.

This narrative review has limitations. Current research lacks controlled studies that have properly matched control groups and other variables isolated, such as equal access to performance-optimizing food options. Other studies investigate changes in nutrition knowledge before and after a sports RD presents education material. This is often not followed up to investigate if it translates to improved eating habits or performance outcomes. There is a need for more thoroughly designed studies that evaluate hard endpoints of performance outcomes rather than simply improved knowledge since sports nutrition is focused on optimizing athletic performance as well as improving the knowledge and overall health of the athlete.

## 5. Conclusions

Given the well-documented nutrition risks of student athletes and the observed efficacy of the RD as a nutrition expert and facilitator of improved nutritional outcomes in this population, prospective cohort studies and clinical trials are warranted as the next step in conducting further research with more rigorous methodology. These studies can further explore the nutrition needs of student athletes and how dietitians can impact this population.

## Figures and Tables

**Table 1:** Summary table of articles used demonstrating nutrition knowledge of student athletes.

Title (Author)	Population	Test	Main Outcomes	Significance
Sports Nutrition Knowledge among Mid-Major Division I University Student-Athletes (Andrews et al., 2016) [25]	123 male NCAA D1 athletes	Sports nutrition knowledge questionnaire [12]	90% of participants failed the nutrition knowledge questionnaire, class year, or sport differences between scores	There is a large nutrition knowledge gap in collegiate male athletes.
Sports Nutrition Knowledge of Volleyball Players (Holden et al., 2019) [26]	77 collegiate indoor or sitting Paralympic volleyball players (13 male)	Sports nutrition knowledge questionnaire	Average score of 46% on questionnaire, coaches were the most common nutrition information source (*n* = 51), and no differences in scores between gender, GPA, or diet preferences	General lack of sports nutrition knowledge; non-nutrition trained professionals are common sources for nutrition information.
Nutritional Regrets and Knowledge in National Collegiate Athletic Association Division I Athletes: Establishing a Foundation for Educational Interventions (Madrigal, Wilson, Burnfield, 2016) [27]	196 NCAA D1 athletes from one school (145 male)	Nutrition regret questionnaire and sports nutrition knowledge questionnaire	Females had higher nutritional regrets than male participants (mean rank 112.2 and 90.3, respectively); low median questionnaire scores (48% male and 49% female)	General lack of sports nutrition knowledge; numerous nutritional regrets by males and females.
Dietary intakes and eating habits of college athletes: are female college athletes following the current sports nutrition standards? (Shriver, Betts, Wollenberg, 2013) [28]	52 female NCAA D1 athletes	Nutrition questionnaire (NQ) used for Combined Events Athlete Development project with USA track and field, food logs, and anthropometric measurements	29% of athletes ate less than 3 meals per day, 27% ate less than 2 snacks per day, 73% did not eat regular breakfast, average dining out of 5.4 times weekly and visiting fast food 20% of the times, significant positive correlations between eating out and carbohydrate, and fat intake (*p <* 0.001 both), 58% drank less than 2 cups H_2_O when training, and 56% stated their diet as poor	Poor dietary patterns are common and self-recognized in female athletes.
Nutritional Needs and Attitudes Towards Having a Training Table: Insight form Players from a Division 1 Football Team (Brown, Imthurn, Ramsay, 2015) [29]	77 male NCAA D1 football players	Nutrition questionnaire adapted for this study	75% reported lack of energy during training, 11.8% reported consuming a snack before workouts, 20% consumed electrolyte replacement drinks during workouts, 93.4% reported the potential benefit of a training table, 42% willing to reduce stipend to pay for training table	Collegiate football players do not properly fuel or recover around workouts but see the potential benefit in better nutrition support.
Nutrition practices and knowledge among NCAA Division III football players (Abbey, Wright, Kirkpatrick, 2017) [30]	88 male NCAA DIII football players	Food frequency questionnaire developed for this study, nutrition knowledge questionnaire [12], nutrition course history questions, and 3-day food log	Dined out 2.5 times weekly with 71% fast food consumption. Linemen subgroup ate significantly less fiber (*p =* 0.020), PUFA (*p =* 0.001), Omega-3′s (*p <* 0.001), and Omega 6′s (*p =* 0.001) than the DRI for lineman while eating excess fat (*p =* 0.035), saturated fat (*p =* 0.026), cholesterol (*p =* 0.001), and sodium (*p <* 0.0001). Mean nutrition quiz score of 55.2%, with most participants missing ergogenic aid and micronutrient toxicity questions. Nutrition sources were 25% coaches and 21% non-academic websites with 6% dietitian/nutritionist	Collegiate football players have poor dietary habits and nutrition knowledge. They commonly seek nutrition knowledge from individuals without nutrition training.
Relationship Between Energy Drink Consumption and Nutrition Knowledge in Student-Athletes (Hardy et al., 2017) [31]	194 DIII NCAA athletes (82 male)	General Nutrition Knowledge Questionnaire with energy drink question and demographic questions added	85.5% did not consume energy drinks, mean score of 58.4% on GNKQ, 43% of energy drink users reported negative side effects and energy drink use correlated with poorer questionnaire score (*p =* 0.020)	Energy drink use is low in college DIII athletes, but nutrition knowledge was poor across both groups.
An Investigation of Habitual Dietary Supplement Use Among 557 NCAA Division I Athletes (Barrack et al., 2020) [32]	557 NCAA D1 athletes at southern Californian colleges (298 male)	Supplement use survey	45.2% consumed one or more supplements, 3.6% consumed more than 4 supplements, male supplement users used more supplements (1.2 vs. 0.8 *p =* 0.004) and used more protein/amino acid supplements while females used more vitamin/mineral supplements, males supplemented more often for strength/power, muscle mass and recovery; females supplemented for health. Males were more likely to use the internet and coaches as nutrition information sources (31.8% vs. 19.8% and 27.4% vs. 18.2%)	Supplement use is very common in athletes and males are more likely to supplement for performance and seek nutrition information from non-nutrition professionals.

**Table 2:** Summary table of articles used demonstrating efficacy of sports dietitians.

Title (Author)	Population	Intervention	Test	Main Outcomes	Significance
The Effects of a Sports Nutrition Education Intervention on Nutritional Status, Sport Nutrition Knowledge, Body Composition, and Performance during Off Season Training in NCAA Division I Baseball Players (Rossi et al., 2017) [33]	30 NCAA D1 baseball players	90-min nutritional education session followed by 45-min sessions every 3 weeks for 12 weeks.	3-day food logs, sports nutrition questionnaire, body composition, and performance tests	Energy, protein, and carbohydrate were increased significantly after intervention (*p <* 0.001, *p =* 0.002, *p <* 0.001, respectively) and protein and energy were no longer different than recommendations; only the nutrition intervention group decreased fat mass and body fat % (*p =* 0.014 and *p =* 0.023), intervention group had greater change in 5-10-5 shuttle (*p =* 0.030)	Dietary intervention by an RD can improve proper macronutrient consumption, improve body composition, and have potential performance benefit.
Nutrition education by a Registered Dietitian improves dietary intake and nutrition knowledge of a NCAA female volleyball team (Valliant et al., 2012) [8]	11 NCAA D1 female volleyball players	Individualized nutrition consults with an RD throughout intervention period 4 visits over 4 months	18 food logs, sports nutrition questionnaire	Energy, carbohydrate, and protein increased significantly post-intervention (*p* = 0.002, *p* = 0.010, *p* = 0.010) and ended up closer to recommendations, nutrition knowledge significantly increased (*p* = 0.001)	Dietary intervention by an RD can improve low energy and macronutrient intake and improve nutrition knowledge.
Impact of a Professional Nutrition Program on a Female Cross Country Collegiate Athlete (Syed-Abdul et al., 2018) [34]	Case study of D1 female track and field athlete with energy deficit and menstrual disturbance	1-month professional nutritional program with an RD	DXA scans pre and post with 2, 4, and 16-month follow up scans	Fat mass (2.54 kg to 10.5 kg) and body fat% (4.7% to 10.5%) increased post-intervention and through 16-month follow-up, improved subjective reports on well-being	Professional nutrition programming can help recover an athlete from energy deficit leading towards the athlete triad symptoms.
Availability of a sports dietitian may lead to improved performance and recovery of NCAA division I baseball athletes (Hull et al., 2017) [35]	99 NCAA D1 baseball players	Observation from three universities, 2/3 have RDs with CSSD certification and all have strength coaches without CISSN	Custom nutrition and dietary habit questionnaire	RD university groups consumed less fast-food (*p* = 0.020) and caffeine (*p* = 0.020) on weekdays and soda (*p* = 0.080) on weekends and were more likely to take daily multivitamin (*p* = 0.020) and consume fast food on team trips (*p* = 0.010). RD group also were more likely to eat before workouts (*p* = 0.010), refuel after workouts (*p* = 0.010), and prepare >3 meals weekly (*p* = 0.070)	Access to an RD improves daily eating habits and fueling/refueling around workouts and gamedays for college baseball players.
Sports nutrition knowledge among collegiate athletes, coaches, athletic trainers, and strength and conditioning specialists (Torres-McGehee et al., 2012) [36]	579 participants (185 athletes, 131 coaches, 192 athletic trainers, 71 strength and conditioning specialists (SCS))	Cross-sectional survey	Multiple choice questionnaire developed by sports nutrition professionals	Athletes used SCS 16.2%, athletic trainers 11.4% and coaches 7.7% for nutrition resources with no significant use of an RD. Coaches, athletic trainers, and SCS recommended an RD as the top nutrition resource for athletes. 91% of athletes had inadequate nutrition knowledge while 64.1% of coaches, 28.6% of athletic trainers, and 16.9% of SCS had inadequate knowledge	Athletes consult staff members that are not properly trained in general and sports related nutrition knowledge for nutrition guidance.
Need for and Interest in a Sports Nutrition Mobile Device Application Among Division I Collegiate Athletes (Zuniga et al., 2017) [37]	71 NCAA D1 athletes in southern US (25 male)	Cross-sectional survey	Questionnaire on dietary habits, resources, knowledge, and perceived quality	Most participants used family (59.4%) as top nutrition resource with dietitian at 13.0%. 66.7% said they should eat healthier and 2.8% said they did not need to improve their diet. 77% believe carbohydrate loading is needed for a sprint and 40% did not know glycogen is carbohydrate stored in muscle and liver	Athletes rely on resources who are not properly trained in nutrition; athletes lack basic sports nutrition knowledge.

## Data Availability

All data are reported in Table 1 and Table 2.
